# Green Supercritical CO_2_ Ion-Exchange Strategy for Cation Engineering in Polyheptazine Imides Towards Efficient Photoreduction CO_2_ to C_2_H_4_

**DOI:** 10.3390/nano16080489

**Published:** 2026-04-20

**Authors:** Xin Peng, Lina Du, Gaoliang Fu, Shouren Zhang, Junying Ma

**Affiliations:** 1 School of Chemistry and Chemical Engineering, Henan University of Science and Technology, Luoyang 471000, China; 18781068182@163.com; 2 Henan Provincial Key Laboratory of Nanocomposites and Applications, Institute of Nanostructured Functional Materials, Huanghe Science and Technology College, Zhengzhou 450006, China; fugl@hhstu.edu.cn

**Keywords:** supercritical CO_2_, ion exchange, polyheptazine imide, cation engineering, photocatalytic CO_2_ reduction

## Abstract

Photocatalytic reduction of carbon dioxide (CO_2_) into high-value multicarbon products, such as ethylene (C_2_H_4_), remains a significant challenge due to the difficult C-C coupling process. Potassium poly(heptazine imide) (K-PHI) is a promising photocatalyst, yet efficiently exchanging its interlayer cations to tune catalytic selectivity without causing structural degradation is difficult. Herein, an efficient and green supercritical CO_2_ (SC CO_2_) assisted ion-exchange strategy was developed to successfully prepare a series of mono-/di-/trivalent cation-doped M-PHI photocatalysts (M = H^+^, Na^+^, Sr^+^, Ca^2+^, Co^2+^, Fe^3+^). Systematic characterizations confirmed that the SC-CO_2_ treatment successfully achieved in-depth cation substitution without destroying the intrinsic heptazine framework, effectively regulating the interlayer structure and significantly optimizing the photoelectrochemical charge separation. Among the prepared samples, H-PHI exhibited the optimal photocatalytic CO_2_ reduction performance with an outstanding selectivity toward C_2_H_4_ generation. Under simulated sunlight irradiation for 3 h, the yields of CO, CH_4_, and C_2_H_4_ C_2_H_4_ C_2_H_4_ reached 3564.87, 807.32, and 40.00 μmol·g^−1^, respectively, significantly outperforming pristine K-PHI and other metal-doped samples. Crucially, isotope-tracing experiments utilizing a SC CO_2_-DCl treatment detected deuterated CH_4_ and C_2_H_4_ products, providing direct evidence that the hydrogen in the carbon products originates from the introduced protons, thereby elucidating the precise reaction pathway for C-C coupling. This study provides a green and efficient supercritical CO_2_ ion exchange strategy for the cation engineering of crystalline carbon nitride, and also offers new ideas and methods for designing high-activity photocatalysts for photocatalytic CO_2_ reduction.

## 1. Introduction

Photocatalytic conversion of carbon oxide (CO_2_) into high-added solar fuels has emerged as a promising strategy to address both the energy crisis and environment issues [1,2,3]. While significant progress has been made in generating C_1_ products (such as CO and CH_4_), the reduction of CO_2_ to multicarbon (C_2_) products, particularly ethylene (C_2_H_4_), remains a formidable challenge [4]. C_2_H_4_ is a highly desirable chemical feedstock with immense economic value, yet its generation is severely thermodynamically and kinetically limited by sluggish multi-electron transfer steps and the difficult C-C coupling process [5,6,7]. Therefore, designing advanced photocatalysts capable of enriching active protons to steer the reaction pathway toward efficient C-C coupling is of great significance.

Potassium polyheptazine imide (K-PHI), a crystalline and long-range ordered allotrope of polymeric carbon nitride, stands out as an excellent photocatalyst due to its robust conjugated π structure and abundant surface active sites [8,9,10]. Crucially, the K^+^ ions within its extended in-plane cavities are highly exchangeable, making cation engineering an effective means to regulate its electronic structure and catalytic properties [11]. However, conventional aqueous or organic solvent ion exchange methods have the disadvantages of limited permeability, non-uniform ion exchange, easy cation agglomeration and long reaction time, and it is difficult to realize the micro-regulation of the skeleton structure, which restricts the improvement effect of cation doping on the catalytic performance of PHI [12,13,14]. To overcome these limitations, we introduce supercritical CO_2_ SC CO_2_ as a green reaction medium. Supercritical CO_2_ has both the high permeability of gas and the high solubility of liquid. Its low viscosity and high diffusivity enable it to easily penetrate into the micropores or dense structures of materials to achieve efficient mass transfer and reaction [15,16,17,18]. Meanwhile, the strong swelling effect of supercritical CO_2_ on the polymer skeleton can loosen the arrangement of triazine rings, expand the lattice interlayer distance, provide more active sites for ion exchange, and effectively avoid the agglomeration of cations in the skeleton [19].

In this work, we develop a SC CO_2_-assisted ion-exchange method to achieve deep and uniform cation substitution in PHI. A series of M-PHI photocatalysts were prepared by realizing the efficient exchange of K-PHI with monovalent (H^+^, Na^+^, Sr^+^), divalent (Ca^2+^, Co^2+^) and trivalent (Fe^3+^) cations via the supercritical CO_2_-assisted ion exchange method. Systematic structural and photoelectrochemical characterizations confirmed that the SC CO_2_ treatment achieved deep cation substitution and significantly optimized charge separation while preserving the intrinsic heptazine framework. Remarkably, the protonated sample (H-PHI) exhibits the highest photocatalytic CO_2_ re-duction activity, with a unique and strongly enhanced selectivity toward C_2_H_4_ in addition to CO and CH_4_.

Herein, we demonstrate for the first time that SC CO_2_-assisted cation engineering can overcome the limitations of conventional ion-exchange methods for PHI, enabling deep proton exchange that significantly promotes C–C coupling to ethylene; using a designed SC CO_2_-DCl isotopic labeling experiment [20,21,22], we provide direct evidence that the hydrogen atoms in hydrocarbon products (including C_2_H_4_) originate from the introduced protons, thereby offering mechanistic insight into the proton-driven C–C coupling pathway. These findings not only establish an effective modification strategy for crystalline carbon nitrides but also provide direct experimental evidence for the reaction mechanism of CO_2_ photoreduction toward C_2_ products.

## 2. Materials and Methods

### 2.1. Materials

All chemical reagents were of analytical grade and utilized without additional puri-fication. Melamine (99%, Aladdin reagent Co., Shanghai, China), potassium chloride (99.5%, 3Achem, Anqing, China), po-tassium Thiocyanate (99%, Aladdin reagent Co., Shanghai, China), sodium chloride (AR 99.5%, Aladdin reagent Co., Shanghai, China), trolamine (AR, Aladdin reagent Co., Shanghai, China), hydrochloric acid (37%, Xilong Scientific Co., Ltd., Shantou, China), cobalt(II) acetylacetonate (98%, Aladdin reagent Co., Shanghai, China), sodium ethoxide (98%, Aladdin reagent Co., Shanghai, China), strontium chloride (99.5%, Aladdin reagent Co., Shanghai, China), iron acety-lacetonate (98%, Aladdin reagent Co., Shanghai, China), calcium chloride (98%, Aladdin reagent Co., Shanghai, China), Co-baltous chloride (97%, Aladdin reagent Co., Shanghai, China).

### 2.2. Synthesis of Photocatalyst

#### 2.2.1. Synthesis of K-PHI Photocatalyst

Melamine (C_3_H_6_N_6_) (2 g) and potassium chloride (2 g) were weighed, placed in a quartz agate mortar, and ground to homogeneity. The mixture was then transferred into a covered alumina crucible. Thermal polycondensation was carried out at 550 °C with a heating rate of 5 °C min^−1^ for 3 h. After the reaction, the resulting solid powder was collected, washed four times with ultrapure water, centrifuged, and dried in an oven. After drying, 1.5 g of the obtained solid powder was ground with 3 g of potassium thiocyanate (KSCN) until thoroughly mixed. The mixture was placed back into an alumina crucible and heat-treated under a nitrogen atmosphere. The thermal treatment consisted of two stages: the temperature was first raised to 400 °C at a rate of 30 °C min^−1^ and held for 1 h, then further increased to 550 °C at the same heating rate and maintained for 30 min. After the sample was naturally cooled to room temperature, it was washed repeatedly with deionized water to remove residual salts. Finally, the sample was placed in a vacuum oven at 60 °C overnight to obtain the final K-PHI material.

#### 2.2.2. Synthesis of Cation-Doped Heptazine Imide (M-PHI) Photocatalyst

First, 50 mg of the as-prepared K-PHI was dispersed in 5 mL of ethanol and sonicated for 1 h to form a uniform dispersion. Then, 0.5 mmol of strontium chloride (SrCl_2_·6H_2_O) (0.1333 g) was dissolved in 5 mL of ethanol and magnetically stirred until homogeneous, after which it was added to the as-prepared K-PHI dispersion. The resulting mixture was transferred to a supercritical CO_2_ apparatus for cation exchange reaction. The reaction was conducted at 40 °C and 20 MPa with stirring for 8 h. After naturally cooling to room temperature, the CO_2_ gas was slowly released, and the liquid in the reactor was centrifuged three times to collect the solid sample. The obtained sample was dried in a vacuum oven at 70 °C overnight to afford the desired Sr-PHI catalyst.

For the preparation of other cation-doped PHI catalysts, the following steps were fol-lowed: 0.5 mL of concentrated hydrochloric acid was dissolved in 2.5 mL of water; 0.5 mmol of iron(III) acetylacetonate, 0.5 mmol of cobalt(II) acetylacetonate, 0.5 mmol of so-dium ethoxide, and 0.5 mmol of calcium chloride were each dissolved in 5 mL of ethanol, respectively. Each solution was then added to a uniformly dispersed K-PHI suspension. The remaining reaction procedures were identical to those described in the second step, yielding the corresponding catalysts: H-PHI, Fe-PHI, Co-PHI, Na-PHI, and Ca-PHI.

## 3. Results

### 3.1. Structural Characterization of M-PHI Catalysts Constructed by Supercritical CO_2_-Assisted Ion Exchange

**Figure 1:**
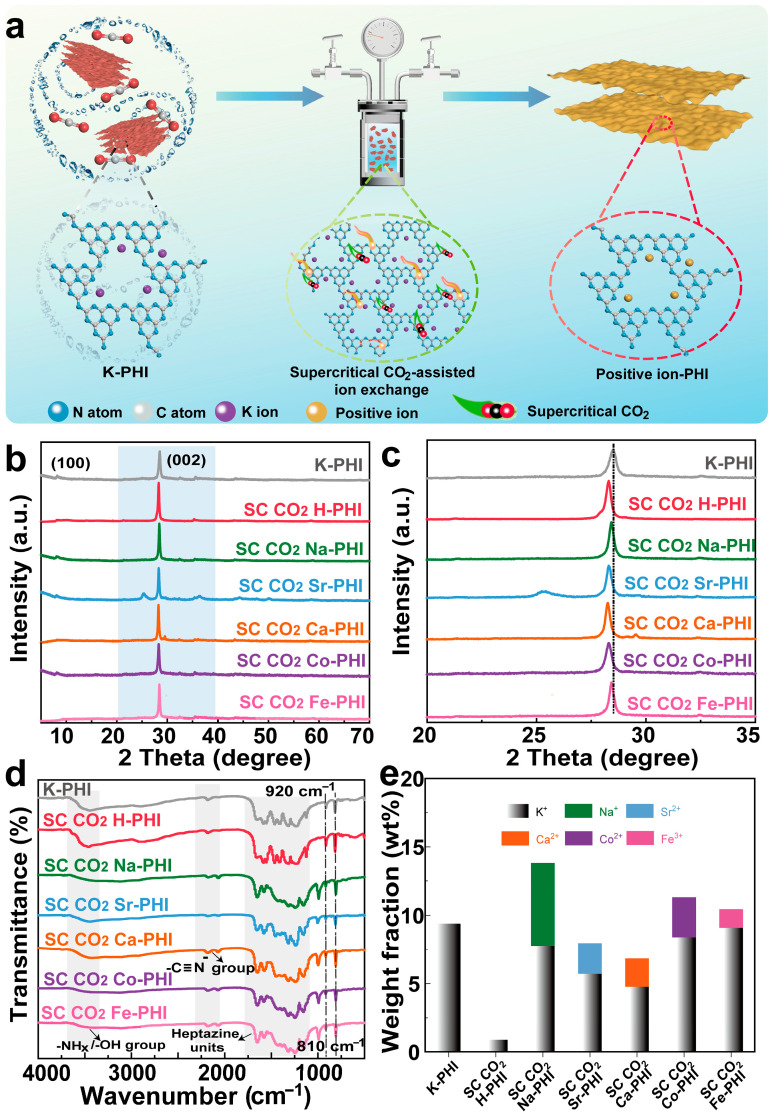
(**a**) Schematic diagram of the process for constructing M-PHI photocatalyst using supercritical CO_2_; (**b**,**c**) XRD spectrum of M-PHI photocatalyst; (**d**) FTIR spectrum of M-PHI photocatalyst; (**e**) ICP test of the metal content of M-PHI photocatalyst.

Figure 1a illustrates the preparation process of M-PHI via the supercritical CO_2_-assisted ion exchange method. Firstly, the highly crystalline K-PHI was synthesized using a traditional KCl molten salt method. Subsequently, the target cations of different valences were introduced into the K-PHI framework. Benefiting from the gas–liquid-like dual characteristics, low viscosity and high diffusivity of supercritical CO_2_, the target cations were efficiently transported deep into the interlayer cavities to uniformly replace K^+^ ions, yielding a series of M-PHI catalysts (SC CO_2_ M-PHI). Compared to conventional aqueous or solvent-based methods, this SC CO_2_ ion exchange strategy significantly shortens the reaction time and ensures uniform ion dispersion, effectively preventing the agglomeration of substituted cations within the PHI framework. Furthermore, the unique swelling effect of SC CO_2_ gently loosens the heptazine skeleton. This non-destructive expansion not only facilitates a more thorough ion-exchange process but also helps construct a porous structure, thereby increasing the specific surface area and exposing more accessible active sites for subsequent photocatalytic reactions.

Figure 1b,c show the X-ray diffraction (XRD) patterns of the pristine K-PHI and the prepared SC CO_2_ M-PHI samples. For K-PHI, a characteristic diffraction peak at 8.2° was observed, corresponding to the (100) plane of the in-plane repeating motifs [23]. However, this diffraction peak disappears in the SC CO_2_ Ca-PHI and SC CO_2_ Fe-PHI, indicating a partial loss of long-range in-plane structural order. This is likely due to the strong coordination and structural distortion induced by these multivalent cations during the exchange process. Furthermore, pristine K-PHI exhibited a prominent peak at around 28.2°, corresponding to the (002) plane derived from the periodic interlayer stacking of the conjugated aromatic rings. In contrast, this characteristic peak shifted to a lower angle of roughly 27.0° for the prepared SC CO_2_ M-PHI samples, accompanied by peak broadening. This noticeable downshift explicitly demonstrates that the cation substitution effectively expanded the lattice interlayer distance, while preserving the fundamental heptazine-based polymeric backbone. Such an expanded interlayer spacing is highly advantageous, as it not only facilitates the efficient interlayer migration of photogenerated charges but also provides enlarged spatial channels for the adsorption and activation of CO_2_ molecules.

Figure 1d shows the FTIR spectra of K-PHI and the prepared SC CO_2_ M-PHI samples. The absorption peak at 810 cm^−1^ and 1100~1500 cm^−1^ were attributed to the breathing vibration and the stretching vibration of the heptazine skeleton, the characteristic peak at 2000~2250 cm^−1^ was the stretching vibration of terminal cyano groups. In addition, the broad absorption band at 3000~3500 cm^−1^ originated from the N-H/O-H stretching vibration. All the prepared SC CO_2_ M-PHI samples retain the characteristic absorption peaks of the heptazine ring, confirming that the introduction of cations with different valences does not destroy the heptazine ring skeleton of PHI [24].

Inductively coupled plasma optical emission spectrometry (ICP) was employed to quantify the metal content in the prepared SC CO_2_ M-PHI samples. As shown in Figure 1e, the K^+^ content in pristine K-PHI was 9.389 wt%. After the SC CO_2_-assisted ion-exchange treatment, part of the K^+^ ions were replaced by mono-, di-, and trivalent cations, resulting in a significant decrease in the residual K^+^ content. Among the investigated cations, monovalent ions with ionic radii and valence states similar to K^+^ exhibit relatively higher substitution efficiency. In contrast, divalent (SC CO_2_ Ca-PHI, SC CO_2_ Co-PHI) and trivalent (SC CO_2_ Fe-PHI) cations show lower exchange efficiency, which can be attributed to their higher charge density and different ionic radii, making it more difficult for them to diffuse into and occupy the interlayer cavities of the heptazine framework. Notably, the SC CO_2_ Fe-PHI sample showed the lowest metal incorporation and the highest residual K^+^ content. These results suggest that the ionic radius and charge density of the cations significantly influence the substitution efficiency of K^+^ under the supercritical CO_2_-assisted ion-exchange conditions.

### 3.2. Photoelectrochemical Characterization of M-PHI Catalysts Constructed by Supercritical CO_2_-Assisted Ion Exchange

**Figure 2:**
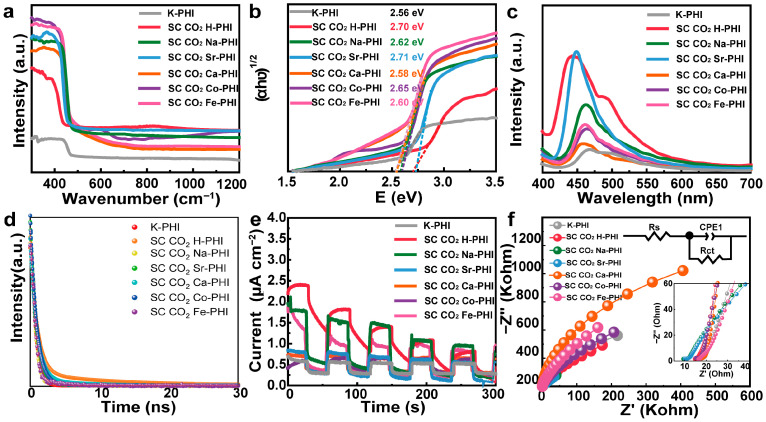
(**a**) Solid UV-vis spectrum; (**b**) Corresponding Tauc’s band gap diagram; (**c**) PL fluorescence spectrum; (**d**) Transient fluorescence lifetime spectrum; (**e**) On-off light response current curve; (**f**) Impedance spectrum (measurement parameters: frequency range from 10^5^ Hz to 0.01 Hz and an Amplitude of 5 mV at the open-circuit potential (OCP).

The optical absorption properties of the samples were investigated by UV-Vis diffuse reflectance spectroscopy (UV-Vis DRS,) as shown in Figure 2a. All sample exhibited a sharp absorption edge at approximately 400~800 nm. The corresponding optical band gaps of the prepared samples were estimated by Tauc plots (Figure 2b) [25,26]. The calculated band gaps of the SC CO_2_ M-PHI sample ranged from 2.58 to 2.71 eV, slightly larger than that of pristine K-PHI (2.56 eV). Among them, SC CO_2_ H-PHI and SC CO_2_ Sr-PHI exhibited relatively wider band gaps, indicating that cation exchange can subtly modulate the electronic structure of PHI. Such band structure modulation may influence the redox ability of photogenerated carriers and subsequently affect the photocatalytic CO_2_ reduction performance.

Photoluminescence (PL) spectroscopy was used to investigate the recombination behavior of photogenerated carriers in the samples (Figure 2c). K-PHI exhibited a strong PL emission peak around 460 nm, indicating a high recombination rate of photogenerated electron-hole pairs [27]. After supercritical CO_2_-assisted ion exchange, the PL intensities of all SC CO_2_ M-PHI samples decreased to varying degrees, suggesting that cation exchange effectively suppresses the recombination of photogenerated carriers. Among the modified samples, SC CO_2_ H-PHI showed the most significant quenching of PL intensity, indicating the most efficient inhibition of charge carrier recombination. In addition, slight shifts in the emission peak positions (around 450–470 nm) were observed for the SC CO_2_ M-PHI samples, which might be associated with changes in the local electronic structure induced by cation substitution. These results demonstrate that the supercritical CO_2_-assisted ion exchange strategy effectively regulates the charge carrier dynamics of K-PHI, thereby facilitating charge separation and potentially enhancing photocatalytic CO_2_ reduction performance.

Time-resolved photoluminescence (TRPL) measurements were further conducted to investigate the charge carrier dynamics of the samples (Figure 2d) [28]. The average lifetime (τ_avg_) of pristine K-PHI is 0.77 ns, indicating rapid recombination of photogenerated carriers. After supercritical CO_2_-assisted ion exchange, noticeable changes in carrier lifetimes were observed. The τ_avg_ values of the modified samples were 5.43 ns for SC CO_2_ H-PHI, 0.85 ns for SC CO_2_ Sr-PHI, 2.994 ns for SC CO_2_ Ca-PHI, 0.74 ns for SC CO_2_ Na-PHI, 0.90 ns for SC CO_2_ Co-PHI, and 0.75 ns for SC CO_2_ Fe-PHI (Table S1). Among them, SC CO_2_ H-PHI exhibited the longest carrier lifetime, which was nearly seven times that of pristine K-PHI. This result indicates that protonation via the supercritical CO_2_ ion-exchange process effectively suppresses the recombination of photogenerated electron–hole pairs and prolongs the carrier lifetime, thereby facilitating charge separation during the photocatalytic reaction.

Transient photocurrent response and electrochemical impedance spectroscopy (EIS) were further employed to evaluate the charge separation and interfacial charge transfer behavior of the prepared SC CO_2_ M-PHI (Figure 2e,f) [29,30]. As shown in the transient photocurrent curves, SC CO_2_ H-PHI exhibited a photocurrent intensity approximately two times higher than that of K-PHI, indicating that the supercritical CO_2_-assisted ion exchange enhances the separation efficiency of electron-hole pairs. The Nyquist plots of EIS showed that the SC CO_2_ H-PHI, SC CO_2_ Na-PHI and SC CO_2_ Sr-PHI exhibited the lowest interfacial resistance, indicating more efficient charge separation and migration. These results collectively suggest that the supercritical CO_2_-assisted cation exchange effectively improves the photoelectrochemical properties of PHI, while protonation modification plays a particularly important role in promoting carrier separation and facilitating charge transfer during photocatalytic CO_2_ reduction.

### 3.3. Band Structure Characterization of M-PHI Catalysts Constructed by Supercritical CO_2_-Assisted Ion Exchange

**Figure 3:**
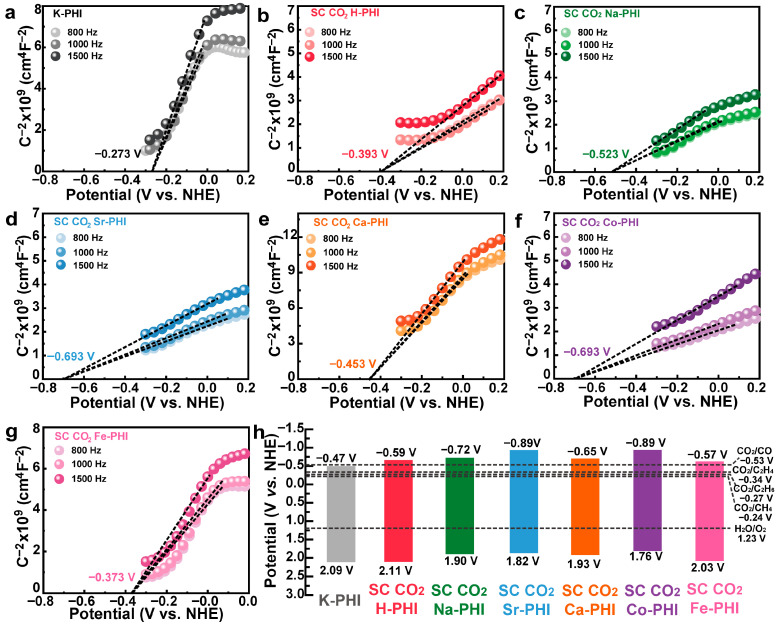
(**a**–**g**) Mott–Schottky curves of the SC CO_2_ M-PHI catalyst (**a**) K-PHI; (**b**) SC CO_2_ H-PHI; (**c**) SC CO_2_ Na-PHI; (**d**) SC CO_2_ Sr-PHI; (**e**) SC CO_2_ Ca-PHI; (**f**) SC CO_2_ Co-PHI; (**g**) SC CO_2_ Fe-PHI; (**h**) band structure diagram.

Figure 3a–g present the Mott-Schottky (M-S) curves of K-PHI and SC CO_2_ M-PHI samples. All samples exhibited positive slopes, confirming n-type semiconductors. The flat band potentials relative to the normal hydrogen electrode (NHE) calculated from the intercepts of the M-S curves are as follows: K-PHI (−0.273 V), SC CO_2_ H-PHI (−0.393 V), SC CO_2_ Na-PHI (−0.523 V), SC CO_2_ Sr-PHI (−0.693 V), SC CO_2_ Ca-PHI (−0.453 V), SC CO_2_ Co-PHI (−0.693 V) and SC CO_2_ Fe-PHI (−0.373 V). Based on the band gap and the flat band potentials derived from the M-S plots, the band structure diagram of the samples was constructed (Figure 3h). The CBM positions of all samples are more negative than the reduction potentials of CO_2_/CH_4_ (−0.27 V vs. NHE) and CO_2_/CO (−0.53 V vs. NHE), indicating that these photocatalysts possess sufficient thermodynamic driving force for the photocatalytic reduction of CO_2_ to CO, CH_4_ and C_2_H_4_. These results demonstrate that cation exchange via the supercritical CO_2_ strategy effectively tunes the electronic band structure of PHI, which is expected to influence the photocatalytic CO_2_ reduction behavior.

### 3.4. Photocatalytic CO_2_ Reduction Performance

**Figure 4:**
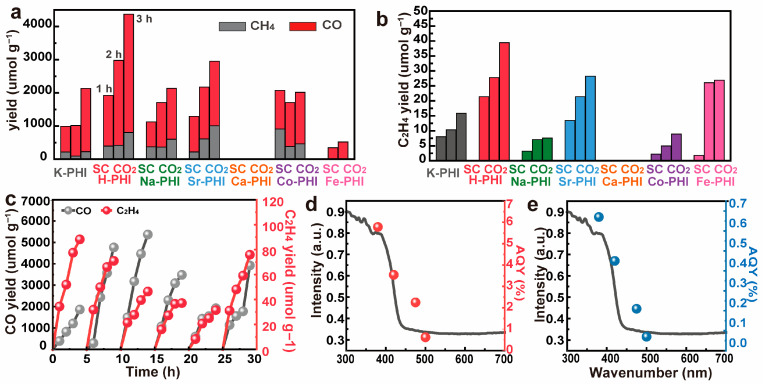
Construction of M-PHI Catalyst for CO_2_ Photoreduction by Supercritical CO_2_ Ion Exchange Method (**a**,**b**) Plot of the yield of CH_4_, CO and C_2_H_4_ as products of M-PHI photo-reduction of CO_2_ in (**a**,**b**) supercritical CO_2_; (**c**) Product diagram of the photocatalytic cycle of H-PHI photocatalyst; (**d**) Apparent quantum yield (AQY) of CO generated by H-PHI photocatalyst; (**e**) Apparent quantum yield (AQY) of C_2_H_4_ generated by H-PHI photocatalyst.

The photocatalytic CO_2_ reduction performance of the catalysts was evaluated in a gas–solid two-phase reaction system, and the results are presented in Figure 4a. Among the investigated samples, SC CO_2_ H-PHI exhibits the highest catalytic activity, with CH_4_ and CO yields reaching 807.32 μmol·g^−1^ and 3564.87 μmol·g^−1^, respectively, after 3 h of simulated sunlight irradiation. In comparison, SC CO_2_ Na-PHI and SC CO_2_ Sr-PHI show moderate production of CH_4_ and CO, whereas no detectable CH_4_ or CO formation is observed for SC CO_2_ Ca-PHI. Overall, photocatalysts doped with monovalent cations (SC CO_2_ H-PHI, SC CO_2_ Na-PHI, SC CO_2_ Sr-PHI) exhibit significantly higher CO_2_ reduction activity than those doped with divalent (SC CO_2_ Ca-PHI, SC CO_2_ CO-PHI) and trivalent (SC CO_2_ Fe-PHI) cations, suggesting that the valence state and ionic characteristics of the introduced cations play an important role in regulating the photocatalytic activity of PHI. Figure 4b further compared the formation of the C_2_ product (C_2_H_4_). Consistent with the trend observed for C_1_ products, SC CO_2_ H-PHI exhibits the highest C_2_H_4_ yield, reaching approximately 40 μmol·g^−1^ after 3 h of photocatalysis. In contrast, the C_2_H_4_ yields of K-PHI, SC CO_2_ Na-PHI, SC CO_2_ Sr-PHI, SC CO_2_ Ca-PHI, SC CO_2_ Co-PHI, and SC CO_2_ Fe-PHI are 15.85, 7.59, 28.26, 0, 8.93, and 26.88 μmol·g^−1^, respectively. Furthermore, we conducted blank experiments under three conditions: without catalyst, without light, and in an Ar atmosphere. The results showed that the yield was 0 in all cases (Table S2).

To evaluate the catalytic stability of SC CO_2_ H-PHI, recycling experiments were performed under identical reaction conditions (Figure 4c). The results showed that after six cycles, the photocatalyst still maintained stable catalytic activity without noticeable deactivation. The yields of CO and C_2_H_4_ remained at 3925.36 μmol·g^−1^ and 75.85 μmol·g^−1^, respectively, demonstrating the excellent durability of SC CO_2_ H-PHI for continuous photocatalytic CO_2_ reduction. Figure 4d,e present the apparent quantum yields (AQY) of SC CO_2_ H-PHI at different wavelengths. The maximum AQY values for CO and C_2_H_4_ production reach 5.78% and 0.67% at 380 nm, respectively. Notably, the AQY spectrum shows a similar trend to the UV–Vis diffuse reflectance absorption profile of the catalyst, indicating that the photocatalytic activity originates from the photoexcitation of SC CO_2_ H-PHI. These results further confirm the efficient light utilization and stable photocatalytic performance of the catalyst.

**Figure 5:**
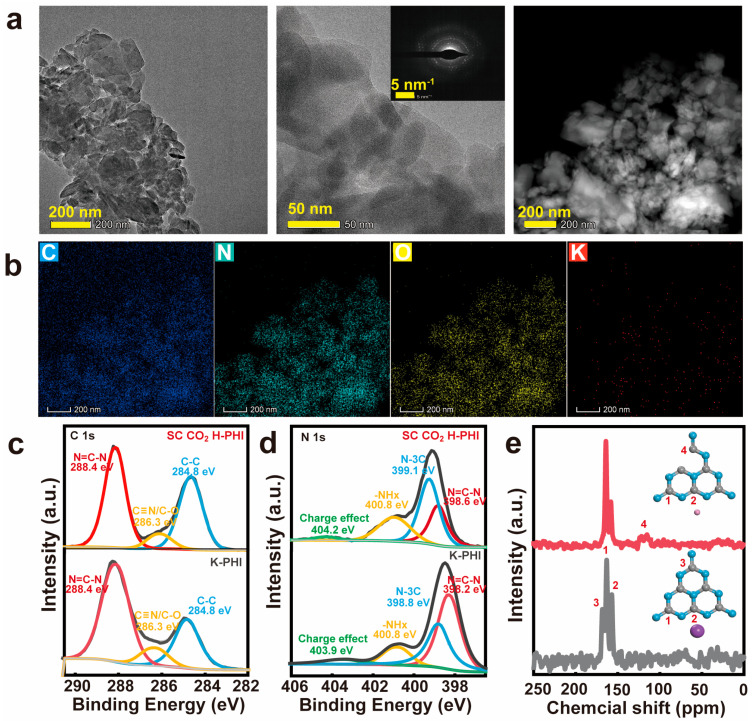
(**a**,**b**) SC CO_2_ H-PHI TEM and its corresponding HADDF image; (**c**) K-PHI and SC CO_2_ H-PHI C 1s spectra; (**d**) K-PHI and SC CO_2_ H-PHI N 1s spectra; (**e**) K-PHI and SC CO_2_ H-PHI solid ^13^C NMR spectra.

Since SC CO_2_ H-PHI exhibited the highest photocatalytic CO_2_ reduction activity, it was selected as a representative sample for further structural characterization in order to understand the origin of its enhanced photocatalytic performance. The SEM and elemental mapping images of the other samples are shown in Figures S1–S6. TEM and HADDF images of SC CO_2_ H-PHI (Figure 5a,b) revealed a typical two-dimensional layered structure with C, N and O elements uniformly dispersed on the stacked nanosheets, while the K element content was extremely low. This observation indicates that the supercritical CO_2_-assisted ion exchange process effectively removes interlayer K^+^ ions and leads to the formation of protonated PHI.

To further investigate the chemical environment of the catalyst, X-ray photoelectron spectroscopy (XPS) analysis was performed. The survey spectra (Table S3) showed that both K-PHI and SC CO_2_ H-PHI consisted mainly of C, N, O elements. Notably, after the supercritical CO_2_ treatment, the content of K 2p decreased significantly from 17.36% to 0%, indicating the complete removal of K^+^ ions from the PHI framework [31]. The high-resolution C 1s spectrum of K-PHI can be deconvoluted into three characteristic peaks located at 284.8 eV, 286.3 eV and 288.0 eV, corresponding to surface defects, edge C≡N groups, and in-ring N–C=N species, respectively (Figure 5c). After the supercritical CO_2_-HCl treatment, the binding energies remained almost unchanged, suggesting that the fundamental heptazine framework was preserved. The N 1s spectrum further provided insight into the structural evolution of PHI. The peaks located at 398.2 eV, 398.8 eV and 400.8 eV corresponding to C–N=C, N–C_3_, and edge –NH_x_ species, respectively (Figure 5d). Compared with K-PHI, the peaks of C–N=C and N–C_3_ shifted slightly to higher binding energies, suggesting a redistribution of electron density in the PHI framework after protonation. Meanwhile, the ratio of C–N=C/N–C_3_ decreased from 0.94 to 0.71 (Table S4), indicating the formation of nitrogen defects during the supercritical CO_2_-HCl treatment. In addition, a weak π–π satellite peak at 404.0 eV is observed, further confirming the modification of the electronic structure of the heptazine units.

Solid-state ^13^C NMR spectroscopy was further employed to investigate the structural evolution of PHI after the supercritical CO_2_ treatment (Figure 5e). For pristine K-PHI, three dominant resonance peaks were observed at approximately 150~160 ppm, corresponding to the sp^2^ carbon atoms in the N–C=N units of the heptazine ring and the bridging carbon atoms in the tri-s-triazine framework, respectively. After the supercritical CO_2_–HCl treatment, SC CO_2_ H-PHI still exhibits the characteristic resonances of the heptazine framework, indicating that the fundamental polymeric structure of PHI is preserved during the ion-exchange process. However, an additional signal located at around 120 ppm becomes more pronounced, which can be attributed to the formation of cyano (-C≡N) groups or edge carbon species [32]. To evaluate the structural robustness of the catalyst, TEM, XRD, and XPS analyses were conducted on the used SC CO_2_ H-PHI sample after the photocatalytic CO_2_ reduction reaction. As shown in Figure S7, the TEM image of the spent catalyst retains the typical lamellar morphology with no observable structural collapse or particle aggregation, indicating good morphological stability. The XRD pattern in Figure S8 displays characteristic diffraction peaks consistent with those of the fresh SC CO_2_ H-PHI, confirming that the crystalline heptazine-based framework remains intact during the reaction. Furthermore, the high-resolution C 1s and N 1s XPS spectra of the used catalyst (Figure S9) exhibit nearly identical binding energies and peak shapes to those of the fresh sample, demonstrating that the chemical environments of carbon and nitrogen species are well preserved. Collectively, these results evidence the outstanding structural, morphological, and chemical stability of the SC CO_2_ H-PHI catalyst under the reaction conditions.

### 3.5. Mechanism Investigation

**Figure 6:**
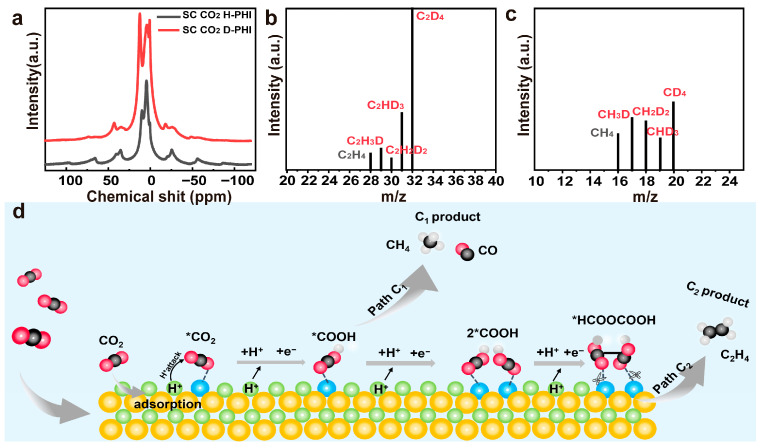
(**a**) ^2^H NMR spectrum of the solid D-PHI catalyst prepared under the supercritical CO_2_-DCl conditions; (**b**,**c**) Isotope gas chromatography-mass spectrometry (GC-MS) analysis of the photo-reduction of CO_2_; (**d**) Schematic illustration of the photocatalytic CO_2_ reduction mechanism over SC CO_2_ H-PHI prepared by supercritical CO_2_-assisted ion exchange. * represents the activated free radical intermediate.

To further clarify the photocatalytic mechanism of SC CO_2_ H-PHI, isotope-labeling experiments were conducted. A deuterated catalyst (SC CO_2_ D-PHI) was prepared using a supercritical CO_2_–DCl system, and the formation of N–D species was verified by solid-state ^2^H MAS NMR spectroscopy (Figure 6a). A clear resonance signal appears in the range of 0~20 ppm, which is characteristic of deuterons bonded to nitrogen atoms. When DCl is used instead of HCl, the intensity of this signal increases markedly, confirming the successful incorporation of deuterium species into the PHI framework. To verify whether these deuterium species participate in the reaction, the photocatalytic products generated by SC CO_2_ D-PHI were analyzed by GC–MS (Figure 6b,c). Besides the normal products C_2_H_4_ and CH_4_, a series of deuterated hydrocarbons including C_2_H_3_D, C_2_H_2_D_2_, C_2_HD_3_, C_2_D_4_ and CH_3_D, CH_2_D_2_, CHD_3_, CD_4_ were detected. The formation of these deuterated products clearly demonstrates that the hydrogen atoms involved in CO_2_ reduction can originate from the surface-adsorbed water and proton species of SC CO_2_ H-PHI. Therefore, SC CO_2_ H-PHI can act as an intrinsic proton reservoir that continuously supplies active hydrogen species during photocatalytic CO_2_ reduction.

Based on the structural and isotopic evidence, the reaction mechanism of the highly productive SC CO_2_ H-PHI is shown in Figure 6d. Upon light irradiation, the CO_2_ molecules were strongly adsorbed and activated on the surface of SC CO_2_ H-PHI. Crucially, the protons (H^+^) anchored within the PHI framework act as an intrinsic proton reservoir. Instead of relying on the sluggish proton transfer from bulk water, these endogenous protons directly and rapidly attack the activated CO_2_ molecules via a highly efficient proton-coupled electron transfer (PCET) process. This localized, abundant proton supply rapidly generates a high surface coverage of *COOH intermediates. Consequently, the physical proximity and high concentration of these intermediates significantly lower the kinetic barrier for the critical C–C coupling step. Adjacent *COOH species readily dimerize to form *HCOOCOOH, which subsequently undergoes continuous reduction and deoxygenation to yield C_2_H_4_. In essence, the synergy between the defect-mediated CO_2_ activation and the endogenous proton supply structurally steers the reaction pathway toward multi-carbon (C_2_) products.

## 4. Conclusions

In summary, a facile supercritical CO_2_-assisted ion exchange strategy is initially developed to construct a series of cation-modified PHI (SC CO_2_ M-PHI) photocatalysts. And the evaluations of photocatalytic CO_2_ reduction performance revealed a striking structure–activity relationship among the prepared variants. Notably, the protonated catalyst (SC CO_2_ H-PHI) stood out, exhibiting remarkably enhanced catalytic activity and selectivity, particularly towards the generation of the C_2_ product (C_2_H_4_). This exceptional performance leap suggests that the SC CO_2_–HCl treatment does not merely alter the physical structure of the framework. More importantly, the successfully introduced H^+^ species transform the PHI framework into an endogenous proton reservoir. Rather than relying solely on the sluggish proton transfer from the external solvent, this self-supplied proton microenvironment is hypothesized to play a pivotal role in accelerating the subsequent proton-coupled electron transfer (PCET) and C–C coupling steps during CO_2_ photoreduction.

## Data Availability

The original contributions presented in this study are included in the article/Supplementary Materials. Further inquiries can be directed to the corresponding authors.

## Supplementary Materials

The following supporting information can be downloaded at: https://www.mdpi.com/article/10.3390/nano16080489/s1.
