# Supplementary material for: Green Supercritical CO2 Ion-Exchange Strategy for Cation Engineering in Polyheptazine Imides Towards Efficient Photoreduction CO2 to C2H4
Source: Nanomaterials (Basel). 2026 Apr 20;16(8):489. doi: 10.3390/nano16080489 (PMC13118860; doi:10.3390/nano16080489)
Supplement: Supplementary file 1 [file nanomaterials-16-00489-s001.zip › nanomaterials-4234281-supplementary.pdf]

Supporting information

Green Supercritical CO_2 Ion-Exchange Strategy for Cation Engineering in Polyheptazine Imides Towards Efficient Photoreduction CO_2 to C_2H_4

Xin Peng [1], Lina Du [2,*],Gaoliang Fu [2], Shouren Zhang [2,*] and Junying Ma [1,*]

[1] School of Chemistry and Chemical Engineering, Henan University of Science and Technology, Luoyang 471000, China

[2] Henan Provincial Key Laboratory of Nanocomposites and Applications, Institute of Nanostructured Functional Materials, Huanghe Science and Technology College, Zhengzhou 450006, China

*Corresponding E-mail: shourenzhang@hhstu.edu.cn; majy379@163.com; dulinna36@hhstu.edu.cn;

Characterization methods

Powder X-Ray diffraction patterns were measured on a Bruker D8 Advance diffractometer equipped with a scintillation counter detector with Cu-Kα radiation (40 kV and 40 mA) applying a 2θ step size of 5° at a scan rate of 1° min^{-1}.

UV-vis-NIR diffuse reflectance spectra was measured by using a UV-3600 Plus UV-Vis-NIR spectrometer (Shimadzu).

Photoluminescence (PL) spectra were recorded at room temperature on a F-4600 spectrofluorometer (380 nm excitation wavelength).

Time-resolved fluorescence spectroscopy was measured using a Edinburgh, FLS980 that contains an LED pulsed light source, monochromator, photomultiplier tube, iris (aperture) for adjusting light level and filter for cutting the excitation light. The K-PHI samples were dispersed in acetonitrile and irradiated using an internal LED at λ = 380 nm. The decay curves were fitted to a double exponential function of the form $\tau_{avg}=(A_1\tau_1^2+A_2\tau_2^2)/(A_1\tau+A_2\tau_2)$, τ_{avg} is the intensity-weighted average lifetime, τ_1 of the first exponential component, τ_2 is the second exponential component, A_1 is the pre-exponential factor for the first component and A_2 is the pre-exponential factor for the second component.

Transmission electron microscopy (TEM) and high-resolution TEM (HRTEM) measurements were obtained on a Japan-JEOL-JEM 2100 F instrument operated at 120 kV.

Scanning Electron Microscopy (SEM) measurements were performed on Hitachi SU-8010 at 3 kV.

Energy Dispersive X-ray Spectrometry (EDS) elemental mapping measurements were obtained on an EDAX genesis instrument at 20 kV.

X-ray photoelectron spectroscopy (XPS) was performed using a Thermo ESCALAB 280 system with Al/K (photon energy = 1486.6 eV) anode mono-X-ray source. All binding energies were calibrated by using the contaminant carbon (C 1*s* = 284.8 eV) as a reference.

Fourier transform infrared (FTIR) spectra were obtained on a Nicolet Nexus spectrometer.

Electrochemical measurements were carried out with a CHI 760E electrochemical testing station with the N_2 inlet and magnetic stir bar. Glassy carbon was used as a WE, Ag wire in $AgNO_3$ with tetrabutylammonium perchlorate (0.1 M) in DCE as a RE, with Pt wire as a counter electrode. Measurements were performed at room temperature (20-25 °C). A solution of tetrabutylammonium perchlorate (0.1 M) in DCE was used as electrolyte. The electrochemical cell was placed in the grounded Faraday's cage in order to reduce noise.

^{13}C solid-state NMR was measured using the Bruker AVANCE III 400 MHz.

Inductively coupled plasma optical emission spectroscopy (ICP-OES) was measured using the Thermo iCAP™ 7200.

The photocatalytic CO_2 reduction measurements were conducted on a sealed off-line reactor (Beijing China Education Au-light). In the CO_2 photocatalytic process, 5 mg of the cation-doped polyheptazine imide catalysts was dispersed in 2 mL ethanol

via 1 h of ultrasonication to ensure a uniform dispersion. Subsequently, the dispersion was dropped into the reactor and dried into a film in an oven. Then the entire container was vacuum-treated to form a sealed state. Under dark conditions, CO_2 gas was introduced for 30 minutes, followed by a manual sample injection. After that, the Xe lamp was turned on for light irradiation, and samples were collected every 1 hour to obtain precipitation amounts of CH_4, CO and C_2H_4. Light irradiation was provided by a Xe lamp (Beijing China Education Au-light) with a standard AM 1.5G filter, outputting the light density of about 100 mW/cm^2, calibrated by a CEL-NP2000 Optical Power Meter (Beijing China Education Au-light Co., Ltd.). The distance from the lamp to the sample was about 10 cm. The evolved gas products (CH_4, CO and C_2H_4) were qualitatively and quantitatively examined by Fuli GC-9790II gas chromatograph equipped with Hayesep A chromatographic column, thermal conductivity detector (TCD) and flame ionization detector (FID) while ultrahigh-purity argon was used as a carrier gas. When a new catalytic cycle began, the reactor was pumped and refilled with CO_2 gas and 0.5 mL ultrapure water.

Figures of Supplementary Material Characterizations

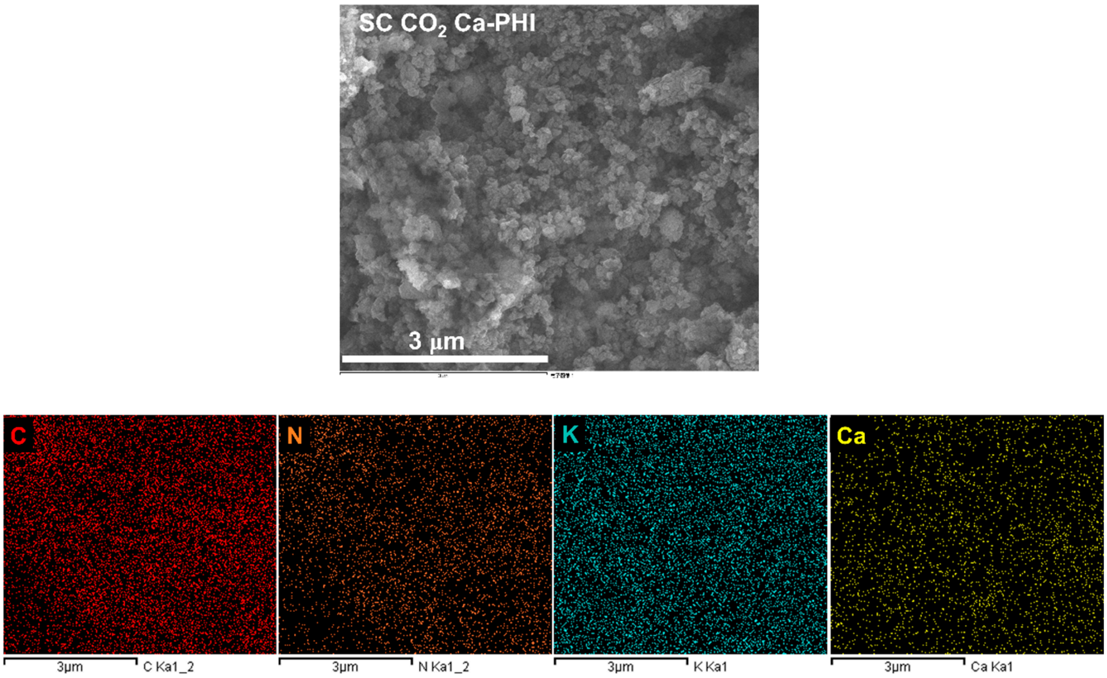

Figure S1. SEM and elemental mapping images of SC CO_2 Ca-PHI.

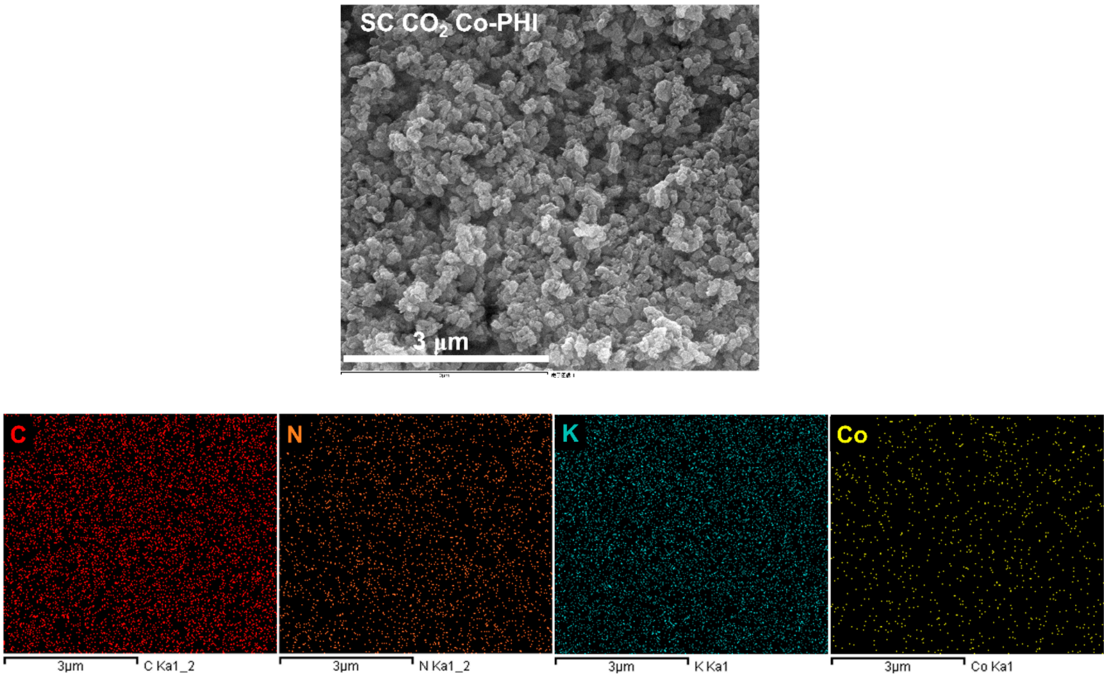

Figure S2. SEM and elemental mapping images of SC CO_2 Co-PHI.

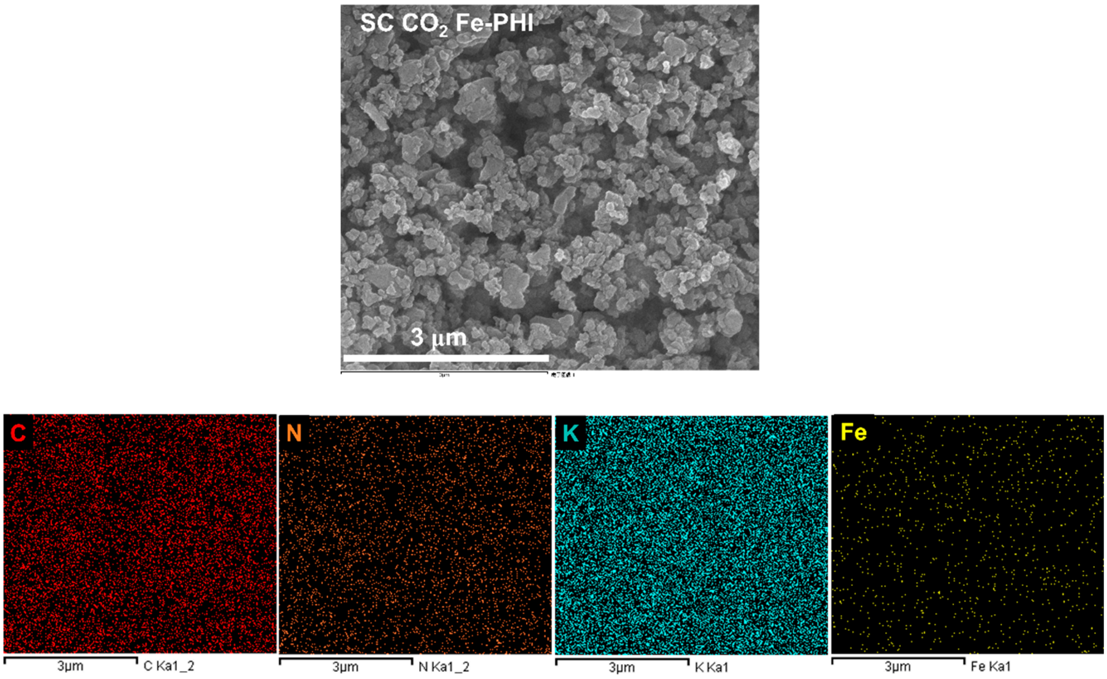

Figure S3. SEM and elemental mapping images of SC CO_2 Fe-PHI.

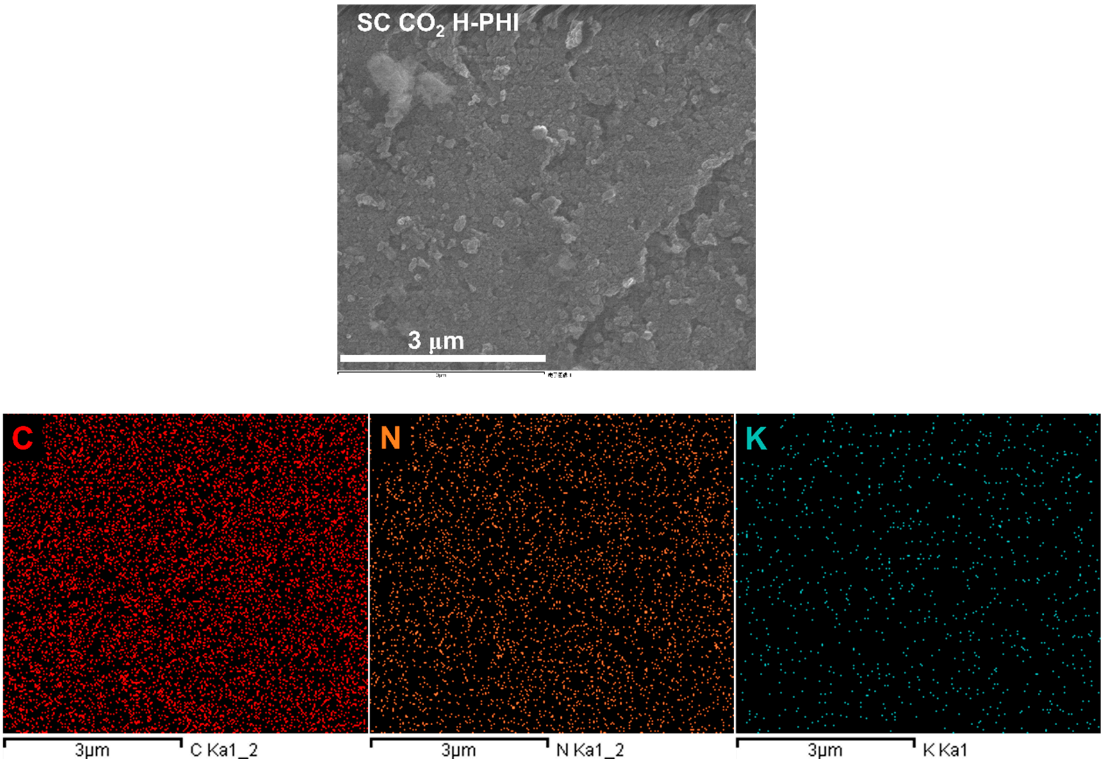

Figure S4. SEM and elemental mapping images of SC CO_2 H-PHI.

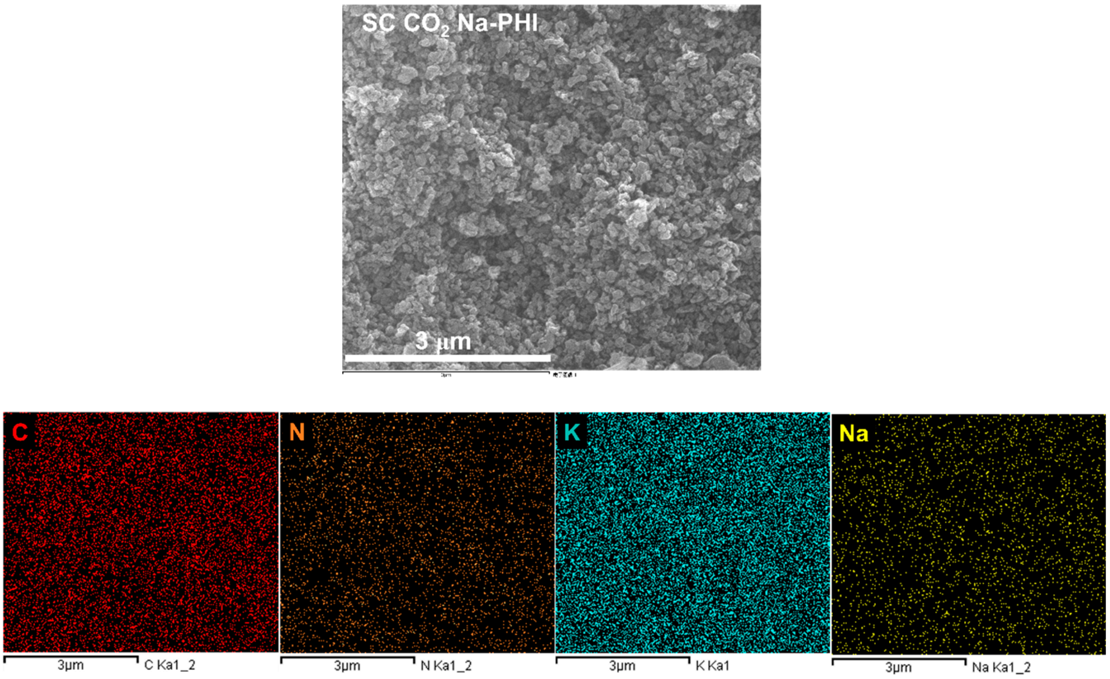

Figure S5. SEM and elemental mapping images of SC CO_2 Na-PHI.

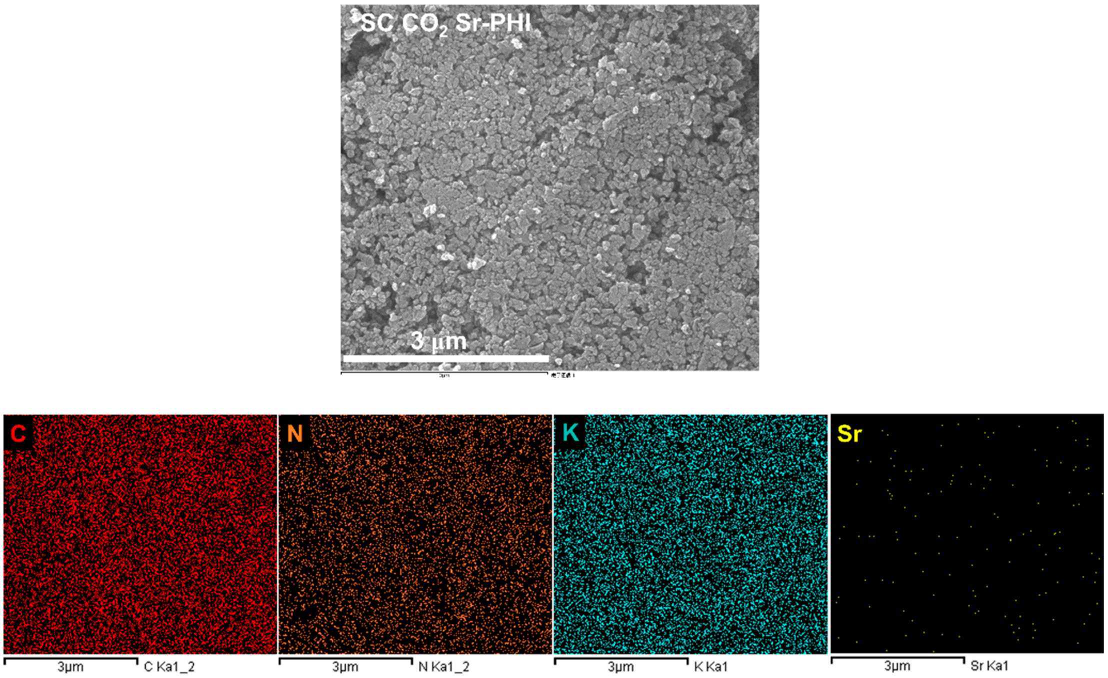

Figure S6. SEM and elemental mapping images of SC CO_2 Sr-PHI.

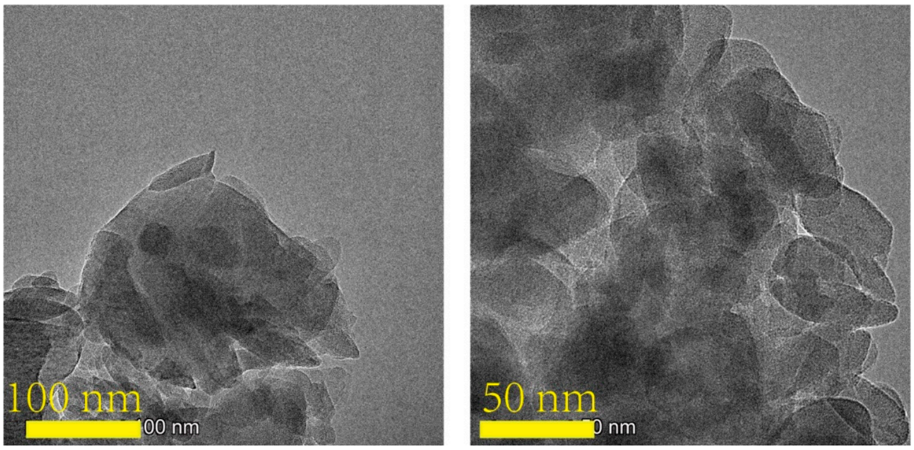

Figure S7. TEM of SC CO_2 H-PHI after reaction.

The TEM image of the spent catalyst retains the typical lamellar morphology with no observable structural collapse or particle aggregation, indicating good morphological stability.

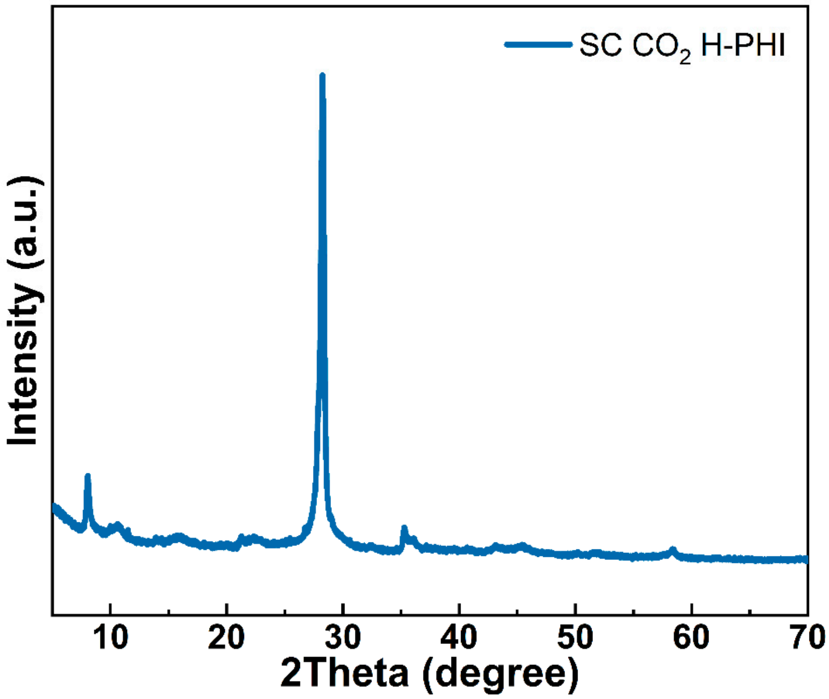

Figure S8. XRD spectrum of SC CO_2 H-PHI after reaction.

The XRD pattern in Figure S8 displays characteristic diffraction peaks consistent with those of the fresh SC CO_2 H-PHI, confirming that the crystalline heptazine-based framework remains intact during the reaction.

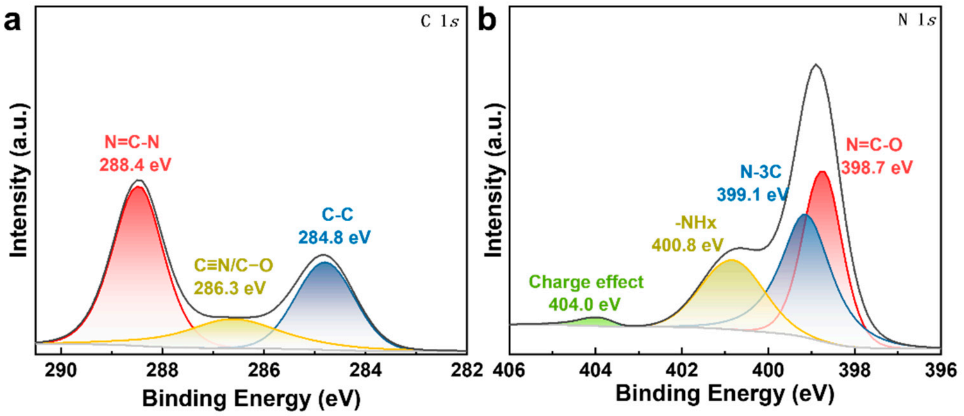

Figure S9. C 1s and N 1s spectra of SC CO_2 H-PHI after reaction.

Furthermore, the high-resolution C 1s and N 1s XPS spectra of the used catalyst (Figure S9) exhibit nearly identical binding energies and peak shapes to those of the fresh sample, demonstrating that the chemical environments of carbon and nitrogen species are well preserved.

Table S1. Average Charge Carrier Lifetimes (τ_{avg}) of Different PHI Samples Determined by Time-Resolved Photoluminescence (TRPL) Measurements.

	τ_1(ns)	τ_2(ns)	τ_{avg}(ns)
K-PHI	0.77	0.77	0.77
SC CO_2 H-PHI	0.96	10	5.43
SC CO_2 Na-PHI	0.74	0.74	0.74
SC CO_2 Sr-PHI	0.85	0.85	0.85
SC CO_2 Ca-PHI	0.95	8.28	2.994
SC CO_2 Co-PHI	0.9	0.9	0.9
SC CO_2 Fe-PHI	0.75	0.75	0.75

Table S2. Photocatalytic CO_2 Reduction Blank Control Experiments.

Experimental Conditions	CH_4 Yield /(μmol·g^{-1})	CO Yield /(μmol·g^{-1})	C_2H_4 Yield /(μmol·g^{-1})
No catalyst, simulated sunlight irradiation, CO_2 atmosphere	0	0	0
With SC CO_2 H-PHI, no light irradiation, CO_2 atmosphere	0	0	0
With SC CO_2 H-PHI, simulated sunlight irradiation, Ar atmosphere (without CO_2)	0	0	0

Table S3. Content of Different Elements in K-PHI and SC CO_2 H-PHI Samples.

	C 1s	N 1s	O 1s	K 2p
K-PHI	23.42	37.76	21.46	17.36
SC CO_2 H-PHI	37.31	37.90	24.79	0

Table S4. Content of Different Components in K-PHI and SC CO_2 H-PHI Samples at N1s.

	C-N=C	N-3C	-NHx	C-N=C/N-3C
K-PHI	38.87	41.39	19.74	0.94
SC CO_2 H-PHI	32.33	45.85	21.83	0.71
